# Ischemic postconditioning confers cerebroprotection by stabilizing VDACs after brain ischemia

**DOI:** 10.1038/s41419-018-1089-5

**Published:** 2018-10-10

**Authors:** Gui-Ying Yao, Qian Zhu, Jing Xia, Feng-Jiao Chen, Ming Huang, Jing Liu, Ting-Ting Zhou, Jian-Feng Wei, Gui-Yun Cui, Kui-Yang Zheng, Xiao-Yu Hou

**Affiliations:** 1 Jiangsu Key Laboratory of Brain Disease Bioinformation, Xuzhou Medical University, Xuzhou, Jiangsu China; 2 Research Center for Biochemistry and Molecular Biology, Xuzhou Medical University, Xuzhou, Jiangsu China; 3 Jiangsu Provincial Key Laboratory of Immunity and Metabolism, Xuzhou Medical University, Xuzhou, Jiangsu China; 4 Department of Neurology, The Affiliated Hospital of Xuzhou Medical University, Xuzhou, Jiangsu China

## Abstract

Ischemic postconditioning provides robust neuroprotection, therefore, determining the molecular events may provide promising targets for stroke treatment. Here, we showed that the expression of functional mitochondrial voltage-dependent anion channel proteins (VDAC1, VDAC2, and VDAC3) reduced in rat vulnerable hippocampal CA1 subfield after global ischemia. Ischemic postconditioning restored VDACs to physiological levels. Stabilized VDACs contributed to the benefits of postconditioning. VDAC1 was required for maintaining neuronal Ca^2+^ buffering capacity. We found that microRNA-7 (miR-7) was responsible for postischemic decline of VDAC1 and VDAC3. Notably, miR-7 was more highly expressed in the peripheral blood of patients with acute ischemic stroke compared to healthy controls. Inhibition of miR-7 attenuated neuronal loss and ATP decline after global ischemia, but also diminished the infarct volume with improved neurological functions after focal ischemia. Thus, ischemic postconditioning protects against mitochondrial damage by stabilizing VDACs. MiR-7 may be a potential therapeutic target for ischemic stroke.

## Introduction

Stroke is one of the leading causes of adult disability and mortality worldwide^1^. Acute ischemic stroke (AIS), caused by systemic hypoperfusion, in situ thrombosis, or embolism, is the most prevalent form of cerebrovascular disease. Early reperfusion, the only widely approved clinical treatment, causes an additional delayed damage to the ischemic brain^2–4^. Much attention has been focused on developing novel neuroprotective strategies for administration after brain ischemia. Ischemic postconditioning, a single or a series of brief interference (s) in the cerebral blood supply performed after a prolonged severe brain ischemia, has been shown to protect against delayed neuronal loss after brain ischemia^5–9^. Thus far, the molecular mechanisms underlying the endogenous neuroprotective effects remain to be defined.

Mitochondria are associated with multiple cellular processes including cell metabolism and cell survival, which implicate mitochondria as having endogenous neuroprotective functions. Voltage-dependent anion channels (VDACs) are the most abundant proteins in the outer mitochondrial membranes (OMM)^10^. The three VDAC isoforms (VDAC1, VDAC2, and VDAC3) are present and share a high structural homology in mammals^11,12^. VDACs, together with the adenine nucleotide translocator 1 (ANT1) in the inner mitochondrial membrane (IMM), function as efficient exchange channels for ATP/ADP. Moreover, VDACs modulate the movement of other small metabolites such as citrate and pyruvate into and out of the mitochondrion and cytoplasm^13^. VDACs have been reported to regulate cancer cell survival by interacting with anchored proteins such as hexokinase 1 (HK1)^14,15^ and Bcl-2 family members^16–19^. Although VDAC1 overexpression has also been implicated in neurodegenerative diseases^20–22^, it is unknown about the contributions of VDACs to ischemic brain damage and underlying molecular mechanisms.

In the present study, we examined the expression of VDACs in the rat brain after global and focal ischemia. We determined whether and how VDAC1, an abundant isoform of VDACs is involved in the prosurvival responses triggered by ischemic postconditioning^23^. We also investigated the molecular mechanisms regulating VDAC expression, which could provide a potential biomarker and therapeutic target for AIS.

## Results

### Ischemic postconditioning prevents VDAC loss in the rat hippocampal CA1 subfield

**Fig. 1:**
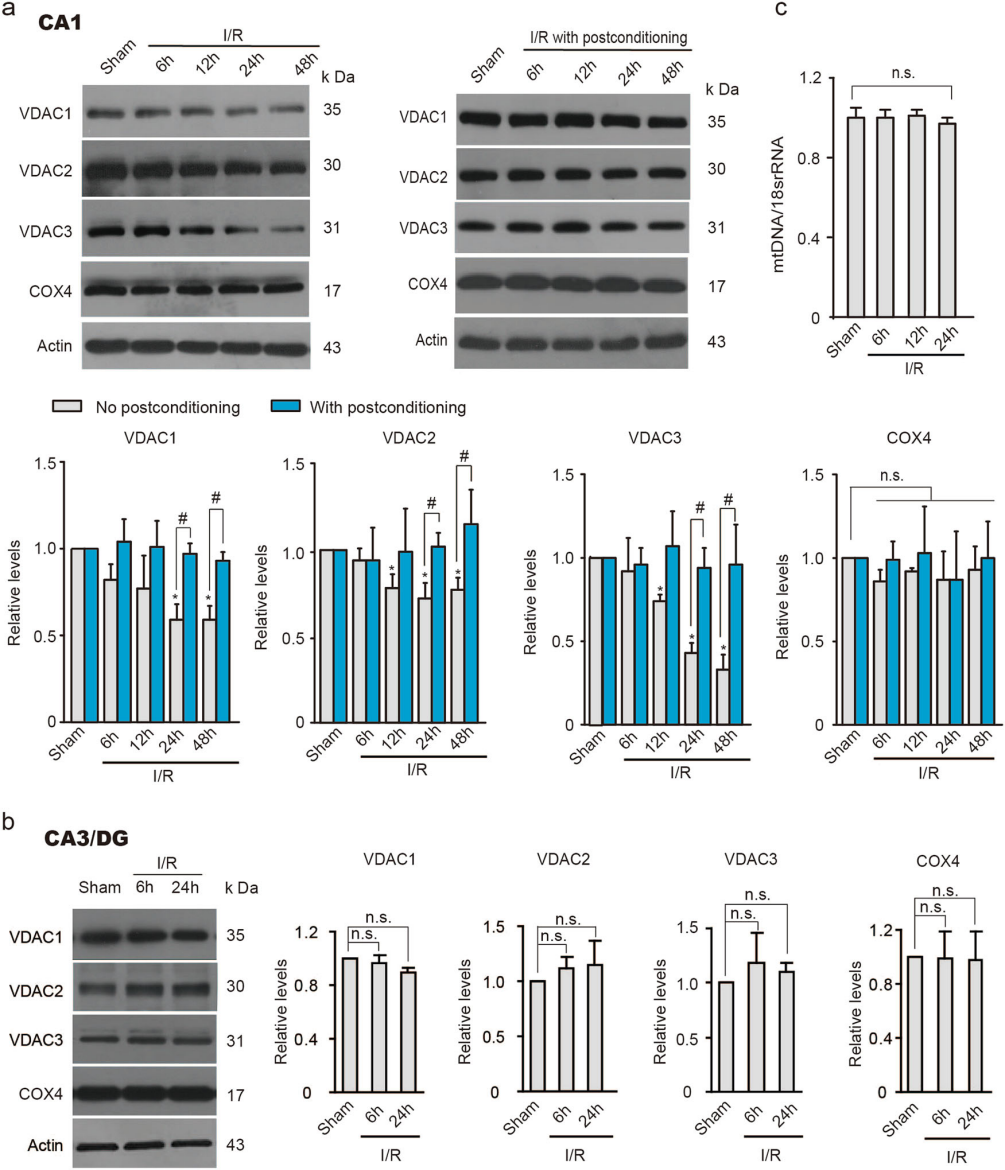
Ischemic postconditioning prevents VDAC decline in rat hippocampal CA1 subfield. **a** and **b** Immunoblots and quantification of VDACs and COX4 after ischemia and reperfusion (I/R) without or with ischemic postconditioning in the hippocampal CA1 subfield (**a**) and in the resistant CA3/DG subfield (**b**) (*n* *=* 3 rats per group); relative levels of VDACs and COX4 were normalized to respective sham groups. Actin was used as a loading control. Data are shown as the mean ± SD of three independent experiments. *P *<* 0.05 versus the sham group; n.s., not significant; One-way ANOVA. ^#^*P* *<* 0.05 versus ischemic postconditioning, unpaired *t*-test. **c** Quantificational analysis of mtDNA copy number in the hippocampal CA1 subfield after I/R (*n* = 3 rats per group); Relative mtDNA copy number was normalized to sham groups. Data are shown as the mean ± SD of three independent experiments. n.s., not significant; one-way ANOVA

**Fig. 2:**
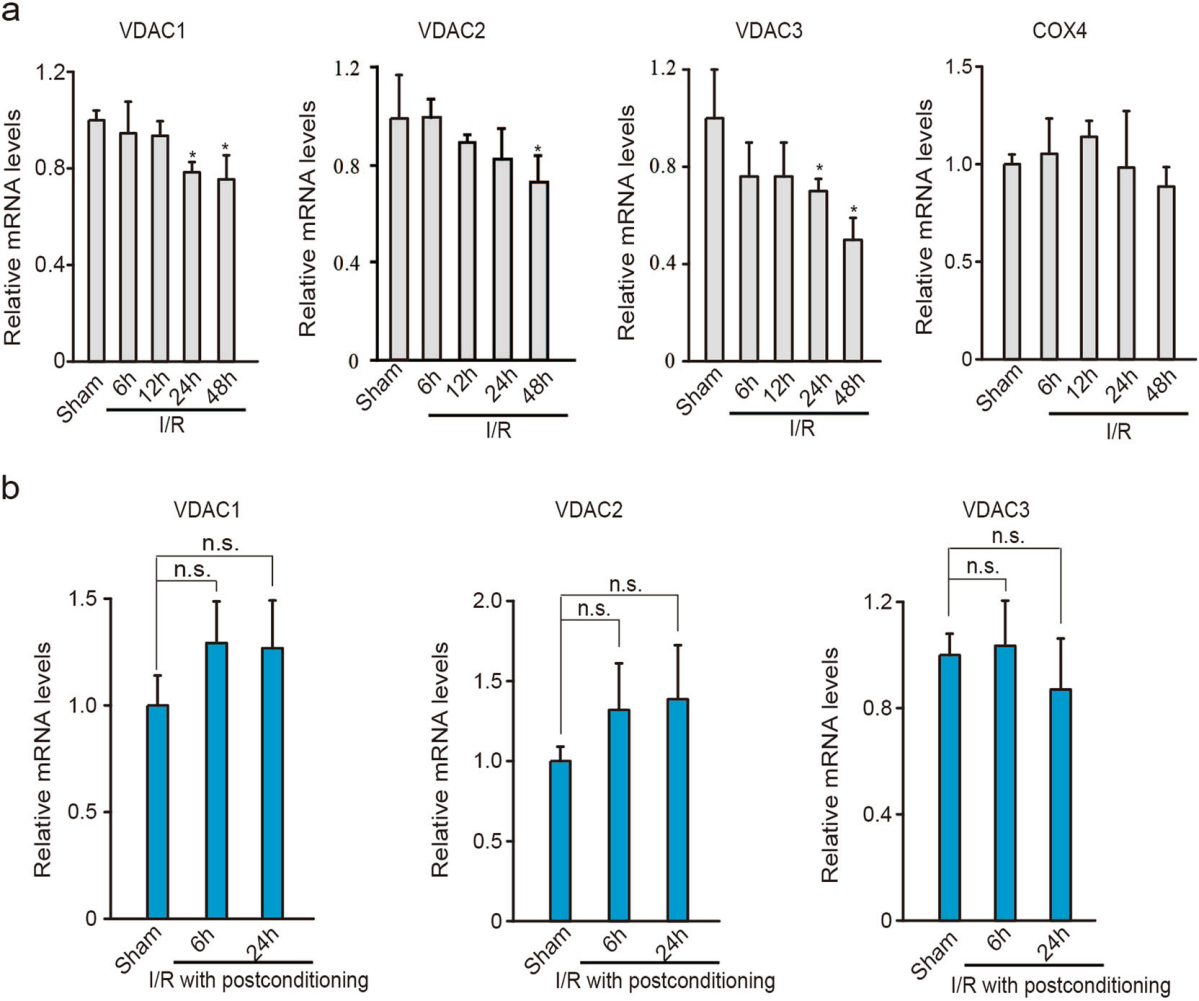
Ischemic postconditioning retains VDAC mRNA expression in rat hippocampal CA1 subfield. **a** and **b** qReal-time PCR analysis of VDAC transcripts in the hippocampal CA1 subfield from rats subjected to ischemia and reperfusion (I/R) without (**a**) or with (**b**) postconditioning (*n* = 3 rats per group for I/R; *n* = 5 rats per group for I/R with postconditioning). Relative levels of VDAC and COX4 mRNA were normalized to respective sham groups. Data represent the mean ± SD. *P *<* 0.05 versus the sham group; n.s., not significant; one-way ANOVA

Multiple reciprocal mechanisms are associated with neuronal susceptibility to ischemia followed by reperfusion, including excitotoxicity, mitochondrial failure, oxidative stress, nitrative stress, and inflammation^24,25^. Previously, we confirmed the efficacy of brief single postconditioning ischemia in a global ischemic model and provided evidence that ischemic postconditioning prevents excitotoxic signaling^8^. To further determine whether ischemic postconditioning confers mitochondrial protection, we measured the expression of the mitochondrial functional VDACs after global ischemia with or without postconditioning. As shown in Fig. 1a, remarkable decreases in all three VDAC isoforms, especially VDAC1 and VDAC3, were observed in rat vulnerable hippocampal CA1 subfield after 15 min of global ischemia followed by reperfusion (I/R). In contrast, I/R did not alter the expression of OMM protein mitofusin 1 in the CA1 subfield (Supplementary Figure S1). Ischemic postconditioning restored all VDAC expressions to the basal levels (Fig. 1a). The expression levels of three VDAC isoforms were not affected in relatively resistant hippocampal CA3/DG subfields after I/R (Fig. 1b). The stable expression of cytochrome c oxidase subunit 4 (COX4) and unaltered mtDNA copy number precluded the possibility of mitochondrial number reduction at the early stage of reperfusion in the CA1 subfield (Fig. 1a–c), which is consistent with previous study^26,27^. Quantitative real-time PCR (qReal-time PCR) analyses showed that mRNA levels of VDACs decreased in CA1 region after ischemia (Fig. 2a), while it was restored to basal levels following ischemic postconditioning treatment (Fig. 2b).

These data suggest that the stable presence of VDACs provides mitochondrial protection and thereafter neuroprotection in CA1 neurons following ischemic postconditioning.

### Stabilized VDAC expression is responsible for the postconditioning-induced neuroprotection

**Fig. 3:**
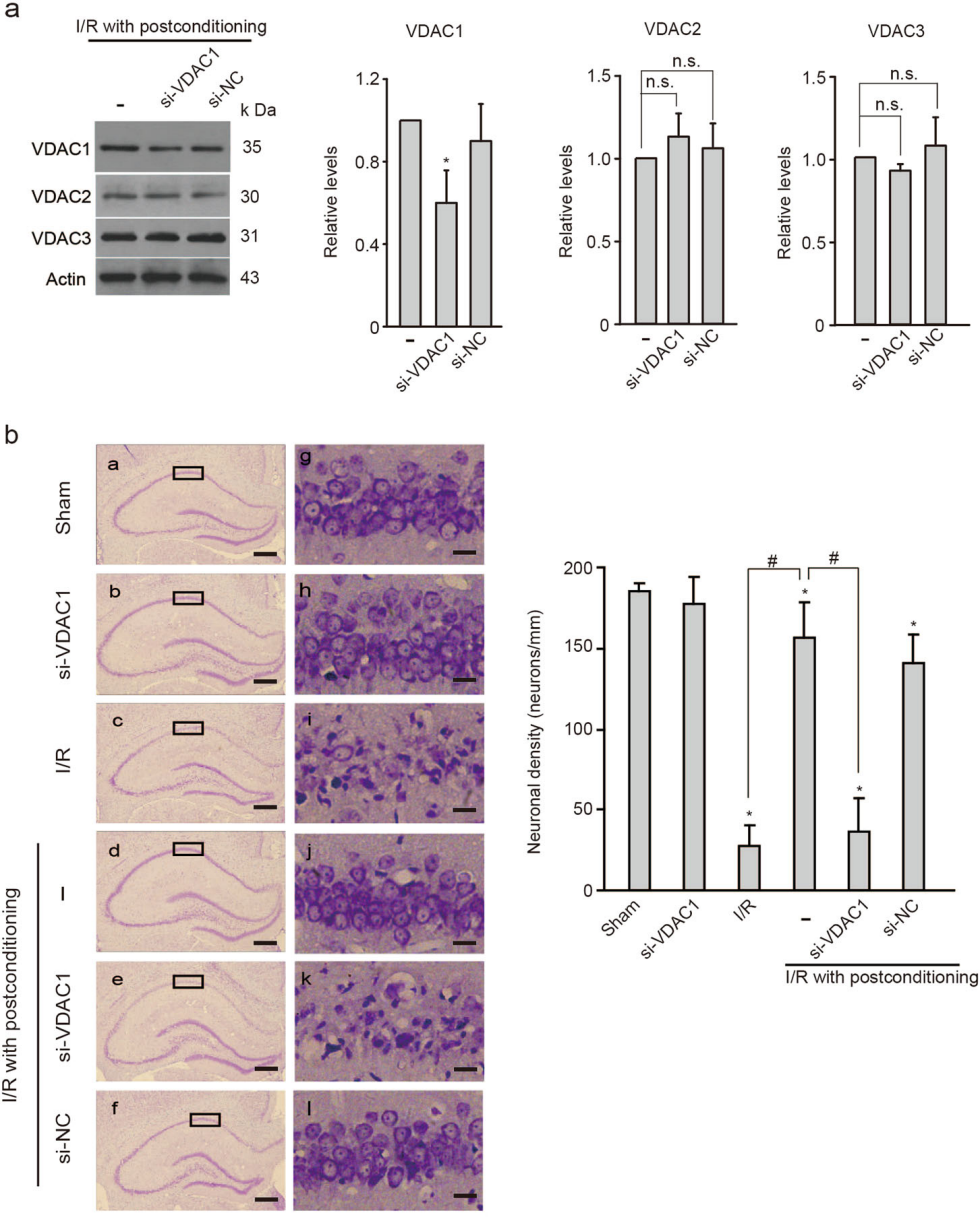
Stabilized VDAC1 contributes to postconditioning-induced neuroprotection. **a** Immunoblots and quantification of VDACs in the hippocampal CA1 subregion 24 h after ischemia and reperfusion (I/R) in rats treated with si-VDAC1 or si-NC (*n* *=* 3 rats per group); Relative levels were normalized to ischemic postconditioning. Actin was used as a loading control. Data represent the mean ± SD of three independent experiments. *P < 0.05 versus I/R with postconditioning group; n.s., not significant; One-way ANOVA. **b** Left, Nissl stained hippocampal sections 5 d after I/R without or with postconditioning in rats treated with si-VDAC1 or si-NC. Scale bar, 500 μm from a to f. Scale bar, 20 μm from g to l. Right, quantification of surviving neurons in the hippocampal CA1 subfield for each group (*n* *=* 5 rats per group). Data are the mean ± SD. *P *<* 0.05 versus the sham group; ^#^*P* *<* 0.05 versus the I/R with postconditioning group; one-way ANOVA

Next, we determined the contribution of VDAC1, the most studied VDAC isoform in rat brain mitochondria, to the postconditioning-induced benefits using siRNA-mediated knockdown. The efficiency and specificity of VDAC1 siRNA (si-VDAC1) were confirmed by immunoblot in rat hippocampus (Fig. 3a). Nissl staining was performed 5 days after I/R to measure the surviving neurons. As shown in Fig. 3b, the ischemic postconditioning group had greater survival of hippocampal CA1 pyramidal neurons compared with those of the I/R group. Conversely, si-VDAC1 delivery abolished the beneficial effect of ischemic postconditioning and triggered severe CA1 pyramidal neuronal loss similar to the I/R group, whereas the negative control sequence of siRNA (si-NC) did not alter neuronal survival (Fig. 3b). These findings confirm that stabilized levels of VDAC1 are required for neuronal survival mediated by postconditioning. Moreover, si-VDAC2 and si-VDAC3 attenuated the postconditioning-induced neuroprotection (Supplementary Figure S2), suggesting that stable presence of VDAC2 and VDAC3 also play important roles in neuronal survival after ischemic stroke.

### Stabilized VDAC1 is required for intracellular calcium homeostasis

**Fig. 4:**
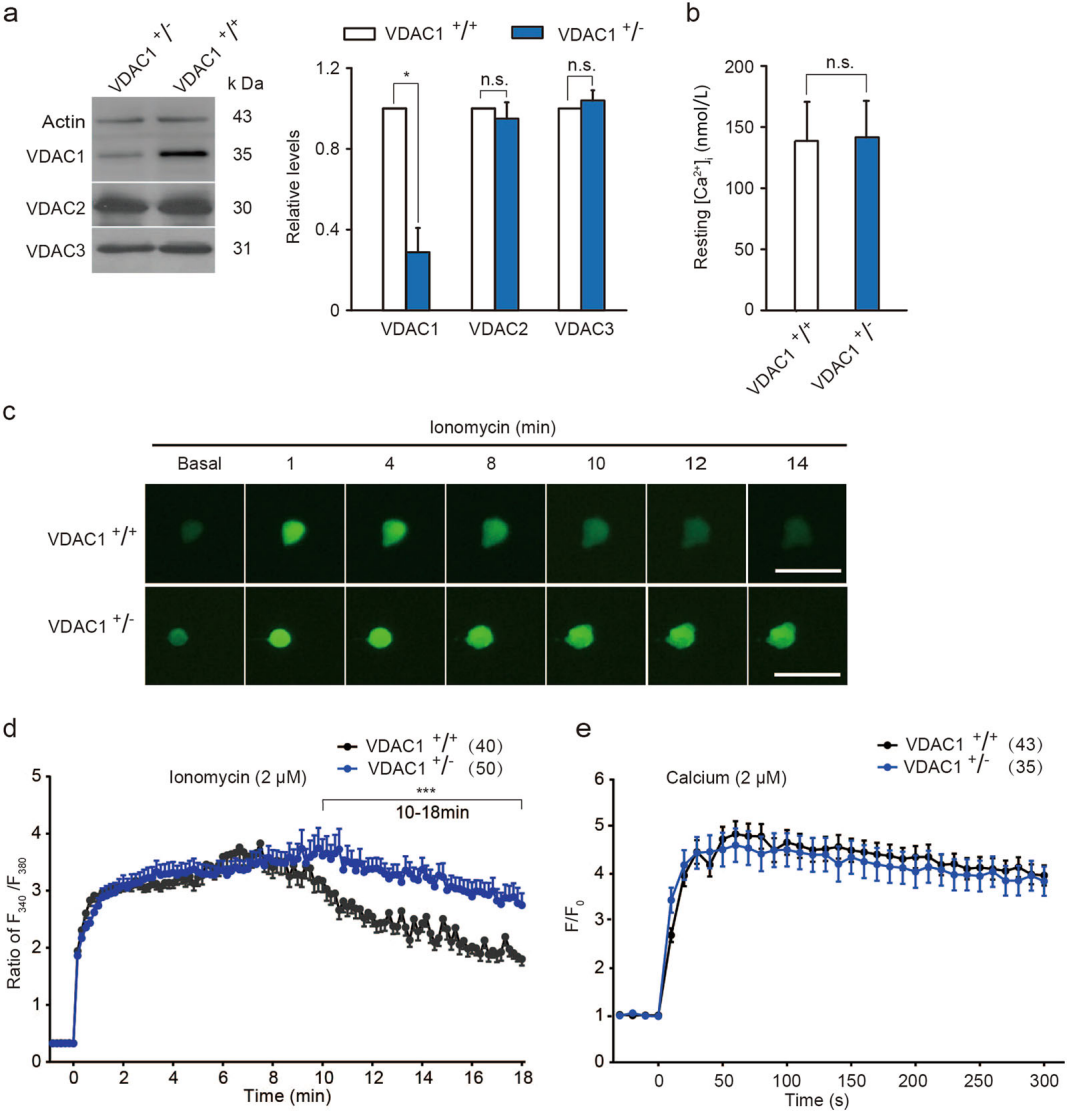
VDAC1 knockdown impairs intracellular calcium buffering capacity. **a** Immunoblots and quantification of VDACs in VDAC1^+/+^ and VDAC1^+/−^ HT22 cells. Relative levels were normalized to VDAC1^+^/^+^ groups (*n* = 3). Actin was used as a loading control. Data are the mean ± SD of three independent experiments. *P *<* 0.05; n.s., not significant; unpaired *t*-test. **b** Resting cytosolic Ca^2+^ levels ([Ca^2+^]_i_) in HT22 cells detected by Fura-2/AM. Data are the mean ± SD (*n* = 39). n.s., not significant; unpaired *t*-test. **c** Time-lapse image of representative calcium fluorescence induced by ionomycin (2 μM) treatment in VDAC1^+/+^ or VDAC1^+/−^ HT22 cells transfected with GCaMP6f plasmid. Scale bar, 50 μm. **d** [Ca^2+^]_i_ fluctuations induced by ionomycin (2 μM) treatment in VDAC1^+/+^ or VDAC1^+/−^ HT22 cells detected by Fura-2/AM. Data represent the mean ± SEM (*n* = 40 for VDAC1^+/+^ HT22 cells; *n* *=* 50 for VDAC1^+/−^ HT22 cells). ***P *<* 0.01 (10–18 min after ionomycin treatment); Two-way ANOVA. **e** Mitochondrial [Ca^2+^] fluctuations in VDAC1^+/+^ or VDAC1^+/−^ HT22 cells detected by Rhod-2/AM under the stimulation of calcium (2 μM). Data represent the mean ± SEM (*n* = 43 for VDAC1^+/+^ HT22 cells; *n* *=* 35 for VDAC1^+/−^ HT22 cells). Two-way ANOVA

The overload of intracellular Ca^2+^ ([Ca^2+^]_i_) is a major cause of delayed neuronal death after brain ischemia^28,29^. To measure the responses associated with VDAC1 deficiency after brain ischemia, an efficient ionophore, ionomycin, was used to elevate the levels of [Ca^2+^]_i_ in HT22 mouse hippocampal cells. Ionomycin elicits a Ca^2+^ influx across the plasma membrane by stimulating store-regulated cation entry^30^. Depletion of VDAC1 had no effect upon resting [Ca^2+^]_i_ in VDAC1^+/−^ HT22 cells comparing with VDAC1^+/+^ cells (Fig. 4a, b). Ionomycin (2 μM) treatment induced a dramatic elevation of [Ca^2+^]_i_ within 1 min in both VDAC1^+/+^ and VDAC1^+/−^ HT22 cells. The [Ca^2+^]_i_ declined 10 min after ionomycin administration in VDAC1^+/+^ HT22 cells (Fig. 4c, d). Under the condition of intracellular calcium overload, no difference was found in intramitochondrial calcium between VDAC1^+/+^ and VDAC1^+/−^ HT22 cells (Fig. 4e).

Caffeine increases [Ca^2+^]_i_ level by calcium-induced calcium release through ryanodine receptors in endoplasmic reticula^31^. Application of caffeine (20 mM)-elicited calcium responses showed a similar tendency with ionomycin in VDAC1^+/+^ or VDAC1^+/−^ HT22 cells (Supplementary Figure S3). These data indicate that VDAC1 deficiency impairs the cellular calcium buffering capacity.

### The downregulation of VDACs is attributed to elevated miR-7

**Fig. 5:**
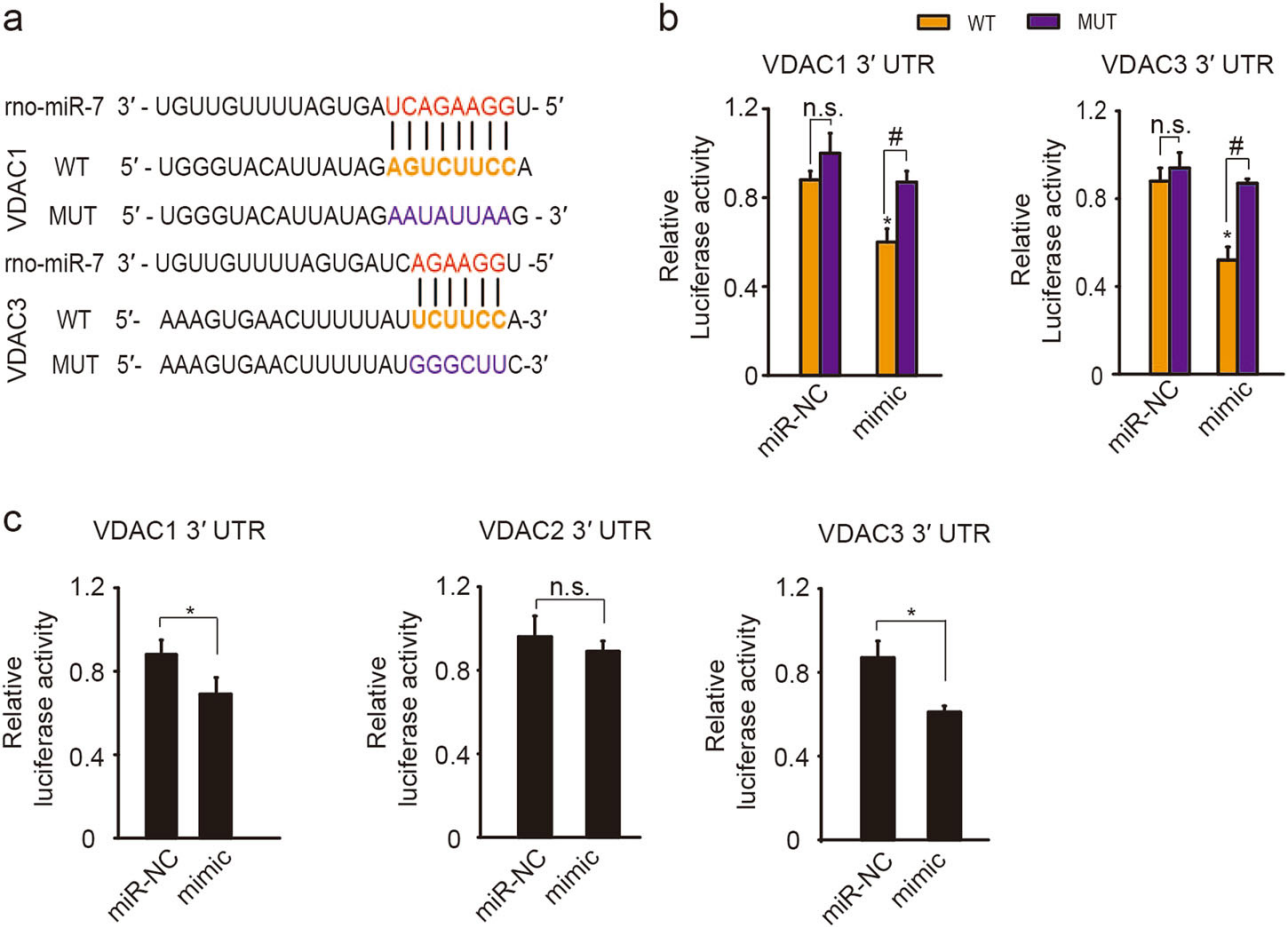
MiR-7 targets VDAC1 and VDAC3. **a** Potential binding sites of rno-miR-7 are shown (colored) targeting wild-type (WT) or mutant (MUT) 3′-UTRs of VDAC1 and VDAC3. **b** Dual-luciferase reporter assays in HEK293 cells transfected with negative control (miR-NC) or miR-7 mimic (mimic) together with 3′-UTRs (WT or MUT) of VDAC1 or VDAC3. Relative luciferase activity was normalized to the group without miR-NC or mimic treatment. Data represent the mean ± SD of three independent experiments. *P *<* 0.05 versus miR-NC group; ^#^*P* *<* 0.05 versus WT group; n.s., not significant; one-way ANOVA. **c** Dual-luciferase reporter assays in HT22 cells transfected with miR-NC or miR-7 mimic together with full-length 3′-UTRs of VDACs. Relative luciferase activity was normalized to the group without miR-NC or mimic treatment. Data represent the mean ± SD of three independent experiments. *P *<* 0.05 versus miR-NC group; n.s., not significant; unpaired *t*-test

Because posttranscriptional and translational regulation may account for the downregulation of VDACs after I/R, we used bioinformatics databases, TargetScan, PicTar, and miRanda, to analyze promising miRNAs that specifically target VDACs. MiR-7 was identified as a potential regulator of both VDAC1 and VDAC3 predicted by all three algorithms. As shown in Fig. 5a, potential miR-7 binding sites were identified in VDAC1 and VDAC3. Dual-luciferase reporter assay was used to examine the direct targets of miR-7 in HEK293 cells. As shown in Fig. 5b, miR-7 mimic, but not the negative control (miR-NC), significantly suppressed the relative luciferase activity from the wild-type (WT) 3′-UTR constructs of VDAC1 or VDAC3. Conversely, the relative luciferase activity from the mutated (MUT) constructs was not decreased by miR-7 mimic (Fig. 5b). Similar inhibitory effects of miR-7 mimic on relative luciferase activity were also found in HT22 cells transfected with full-length 3′-UTR recombinants of VDAC1 or VDAC3, but not VDAC2 (Fig. 5c). The results demonstrate that both VDAC1 and VDAC3 are targets of miR-7.

**Fig. 6:**
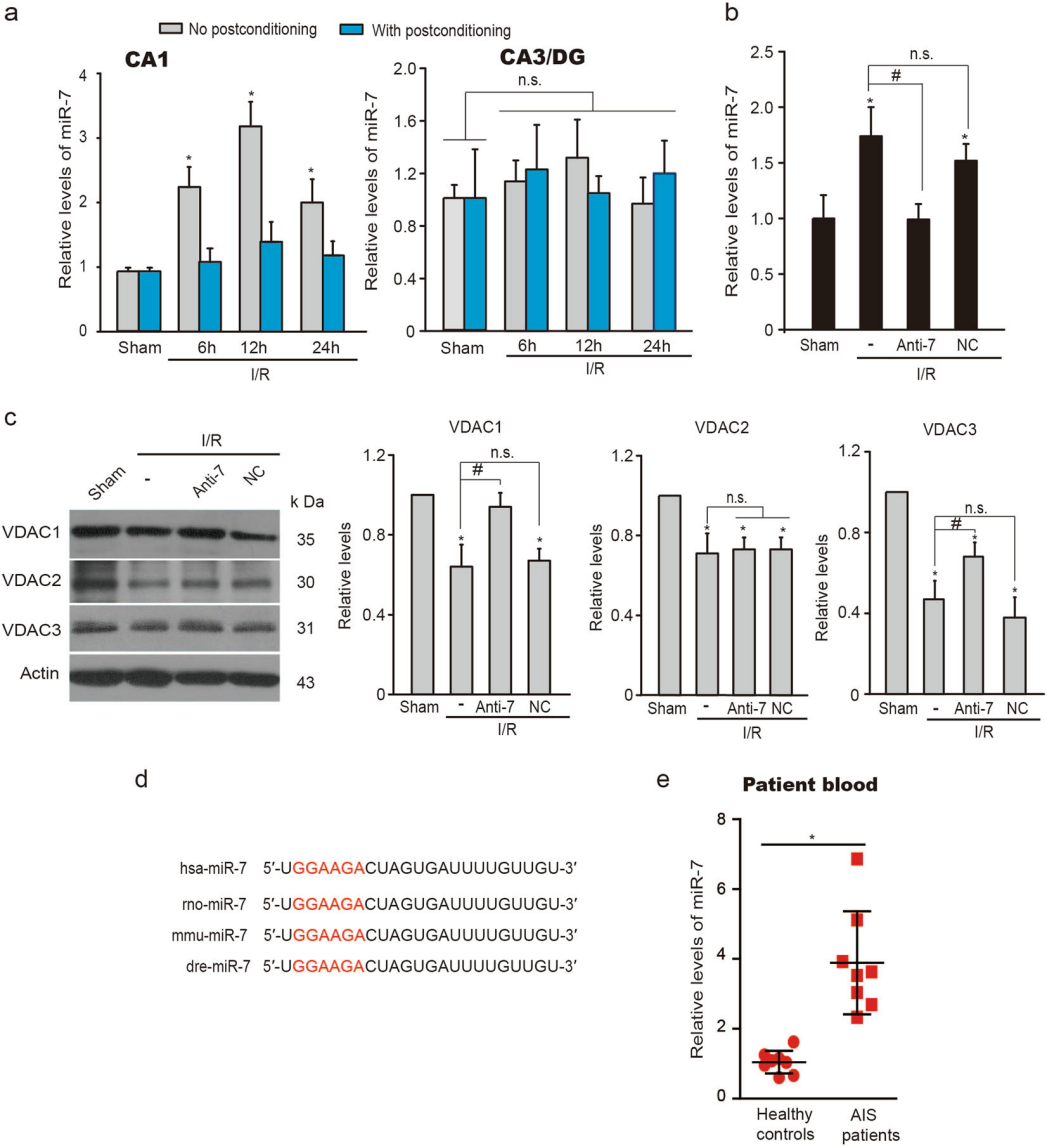
The decrease of VDACs results from elevated miR-7 after ischemia and reperfusion. **a** qReal-time PCR analysis of miR-7 levels in the hippocampal CA1 subfield and in the resistant CA3/DG subfield from rats subjected to global ischemia followed by reperfusion (I/R) without or with postconditioning (*n* = 6 rats per group in CA1 subfield; *n* = 5 rats per group in CA3/DG subfield). Relative levels were normalized to respective sham groups. Data are the mean ± SD. *P *<* 0.05 versus the sham group; n.s., not significant; one-way ANOVA. **b** qReal-time PCR analysis of miR-7 levels in CA1 subfield 24 h after I/R. Rats were treated with Anti-7 or NC 50 min after I/R (*n* *=* 3 rats per group); Relative levels were normalized to sham groups. Data are the mean ± SD. *P *<* 0.05 versus the sham group; ^#^*P* < 0.05 versus the I/R group; n.s., not significant; one-way ANOVA. **c** Immunoblots and quantification of VDACs in the CA1 subfield 24 h after treated with Anti-7 or NC (*n* *=* 3 rats per group); Relative levels were normalized to sham groups. Actin was used as a loading control. Data represent the mean ± SD of three independent experiments. *P *<* 0.05 versus the sham group; ^#^*P* < 0.05 versus the I/R group; n.s., not significant; one-way ANOVA. **d** Conservative analysis of miR-7 sequence in humans, rats, mice, and zebrafish. **e** qReal-time PCR analysis of miR-7 in peripheral venous blood collected within 24 h from patients subjected to acute ischemic stroke (AIS) or healthy controls (*n* = 8 per group). Relative levels were normalized to control groups. Data represent the mean ± SD. *P *<* 0.05 versus the healthy control group; unpaired *t*-test with Welch’s correction

Next, the miR-7 levels were measured in rats. It was shown that miR-7 expression increased significantly in the vulnerable CA1 neurons after I/R, whereas ischemic postconditioning reversed miR-7 expression to the physiological levels (Fig. 6a). No changes were found in the miR-7 levels in the CA3/DG subfield after I/R with or without postconditioning (Fig. 6a). Antisense oligonucleotides specifically against miR-7 (miR-7 inhibitor, Anti-7) attenuated miR-7 expression and upregulated the protein levels of VDAC1 and VDAC3, but not VDAC2, in the hippocampal CA1 region (Fig. 6b, c), suggesting that excessive miR-7 contributes to the postischemic lack of both VDAC1 and VDAC3.

More importantly, miR-7 is of high conservation in rodents and humans (Fig. 6d). The patients with AIS presented markedly elevated miR-7 levels in peripheral blood, when compared with blood from healthy age-matched controls (Fig. 6e), suggesting that circulating miR-7 could serve as a candidate biomarker for AIS.

### Inhibition of miR-7 protects against brain damage after global or focal ischemia

**Fig. 7:**
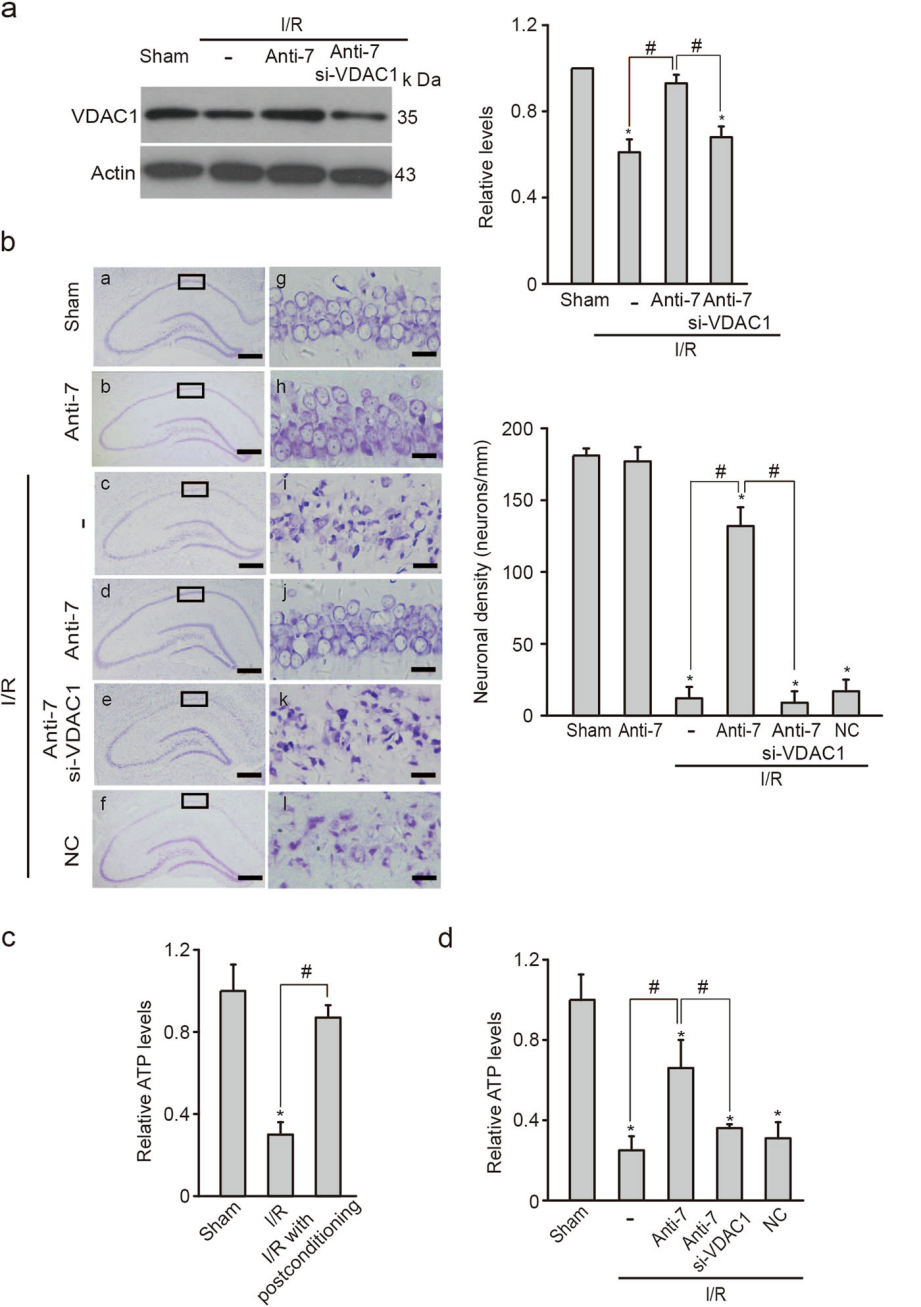
MiR-7 inhibitor exerts neuroprotection against neuronal damage after global ischemia. **a** Immunoblots and quantification of VDAC1 expression in the hippocampal CA1 subregion 24 h after ischemia and reperfusion (I/R) from rats treated with miR-7 inhibitor (Anti-7) together with or without si-VDAC1 (*n* *=* 3 rats per group); Relative levels were normalized to sham groups. Actin was used as a loading control. Data represent the mean ± SD of three independent experiments. *P *<* 0.05 versus the sham group; ^#^*P* *<* 0.05 versus the Anti-7 group; one-way ANOVA. **b** Left, Nissl stained hippocampal sections 5 d after I/R in rats treated with Anti-7 or negative control (NC) together with or without si-VDAC1. Scale bar, 500 μm from a to f. Scale bar, 20 μm from g to l. Right, quantification of the surviving neurons in the hippocampal CA1 subfield for each group (*n* *=* 5 rats per group). Data are the mean ± SD. *P *<* 0.05 versus the sham group; ^#^*P* *<* 0.05 versus the Anti-7 group; one-way ANOVA. **c** Relative ATP levels in hippocampal CA1 region 24 h after I/R (*n* *=* 5 rats per group). Data are shown as the mean ± SD. Relative levels of ATP were relative to sham groups. *P *<* 0.05 versus the sham group; ^#^*P* *<* 0.05 versus the I/R group; one-way ANOVA. **d** Relative ATP levels in CA1 region 24 h after I/R from rats treated with Anti-7 together with or without si-VDAC1 or NC (*n* *=* 5 rats per group). Relative levels of ATP were relative to sham groups. Data are shown as the mean ± SD. *P *<* 0.05 versus the sham group; ^#^*P* *<* 0.05 versus the Anti-7 group; one-way ANOVA

Because VDAC1 is required for functional mitochondria and neuronal survival, targeting miR-7 after ischemia may increase VDAC1 expression and subsequently confer neuroprotection. As expected, Nissl staining showed that Anti-7 delivery after I/R reduced neuronal loss, producing a 77.2% survival rate of CA1 pyramidal neurons, while there was only a 10% survival in the negative control (NC) group (Fig. 7a, b). VDAC1 knockdown by si-VDAC1 abolished the beneficial effect of Anti-7 (Fig. 7a, b).

To explore mitochondrial function associated with Anti-7 neuroprotection, we measured bioenergy genesis and reactive oxygen species (ROS) production in rat hippocampus. As shown in Fig. 7c, d, either postconditioning or Anti-7 treatment restored ATP levels in hippocampal CA1 subfield after I/R, which were eliminated by si-VDAC1. Although postconditioning reduced postischemic ROS level, Anti-7 had no influence on ROS production (Supplementary Figure S4). These data suggest that targeting miR-7 imitates neuroprotection of ischemic postconditioning by increasing VDAC1 expression and ameliorating metabolism impairments.

**Fig. 8:**
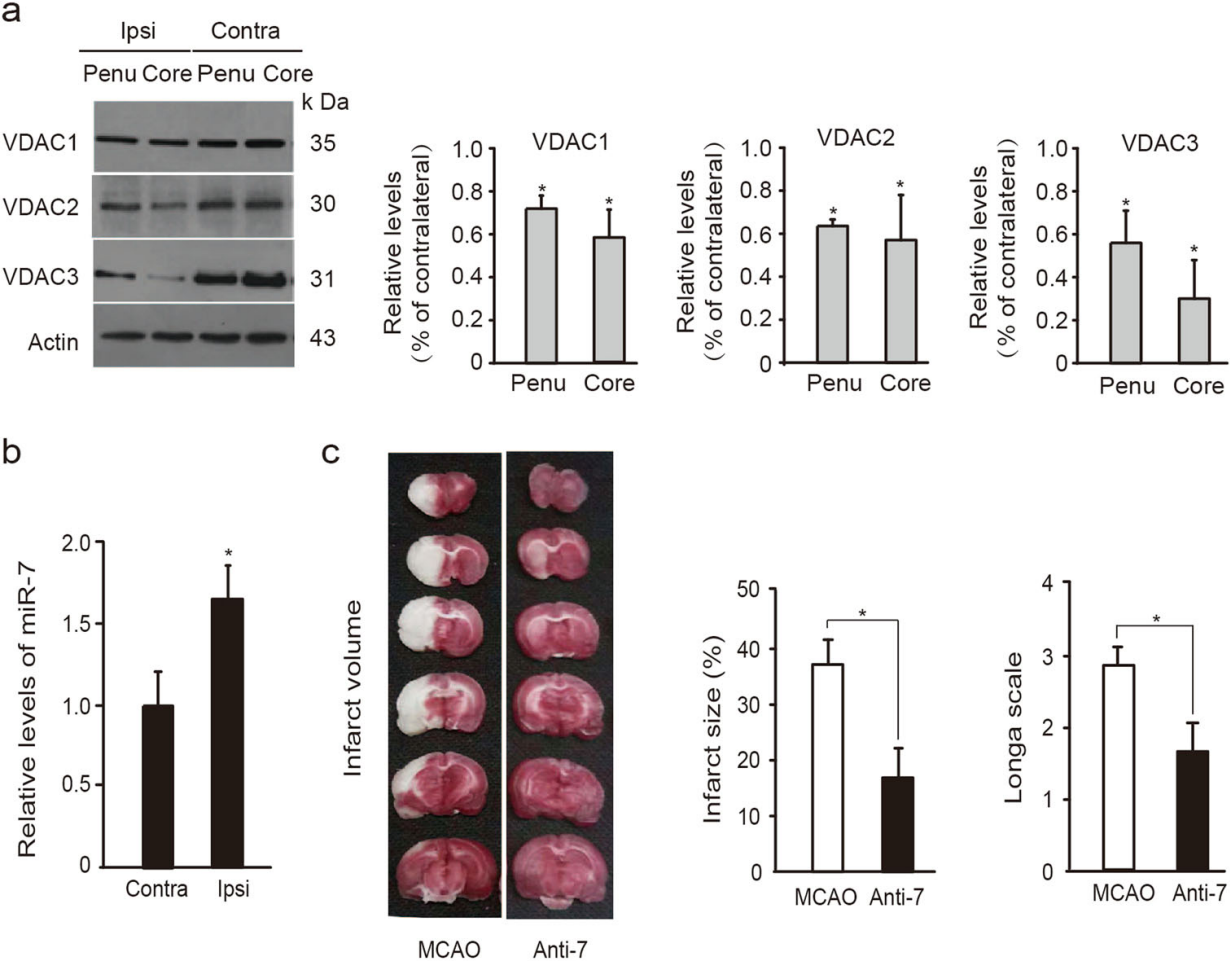
MiR-7 inhibitor exerts neuroprotection against neuronal damage after focal ischemia. **a** Immunoblots and quantification of VDACs in the ischemic ipsilateral (Ipsi) penumbra (Penu), core region (Core), and contralateral normal region (Contra) from rats subjected to MCAO (*n* = 3 rats per group); Relative levels of VDACs were normalized to respective contralateral normal regions. Data represent the mean ± SD of three independent experiments. *P *<* 0.05 versus contralateral region; Student’s *t*-test. **b** qReal-time PCR analysis of miR-7 in penumbra of MCAO rats (*n* *=* 4 rats per group); Relative levels were normalized to contralateral regions. Data are the mean ± SD. *P *<* 0.05 versus contralateral; Student’s *t*-test. **c** Left, TTC-stained brain sections indicating infarct volumes in focal ischemic rats injected with Anti-7 into the cerebral lateral ventricles 40 min after ischemia (*n* = 6 rats each treatment). Middle and Right, quantitative infarct size and neurological deficit in rats were assessed 24 h after MCAO (*n* = 6 rats per treatment). Data are the mean ± SD. *P < 0.05; Student’s *t*-test

Using focal ischemia models of middle cerebral artery occlusion (MCAO), we also found that VDACs were downregulated in the ischemic penumbra regions of the ipsilateral (ischemic) brain hemisphere relative to contralateral regions after I/R (Fig. 8a). Accordingly, miR-7 was highly expressed in the penumbra of the ipsilateral hemisphere (Fig. 8b), suggesting that the miR-7 inhibitor, Anti-7, may display similar beneficial effects against focal ischemia. Indeed, Anti-7 treatment 40 min after MCAO onset diminished the infarct volume, and reduced the neurological deficits when compared with MCAO group (Fig. 8c).

The results suggest that even after ischemia the miR-7 inhibitor mediates neuroprotection against ischemic injury by stabilizing the levels of VDACs.

## Discussion

Although reperfusion following brain ischemia restores the blood supply of oxygen and glucose, mitochondrial dysfunctions make neurons especially vulnerable because of impairments in bioenergy genesis, Ca^2+^ handling, and anti-apoptotic signaling^32,33^. In this study, we provided evidence that the deficiency of OMM VDACs is responsible for mitochondrial dysfunction after ischemic stroke. We also showed that ischemic postconditioning provides mitochondrial protection by preventing excessive decreases of VDAC levels.

All three VDAC isoforms, especially VDAC1 and VDAC3, were downregulated in the susceptible hippocampal CA1 subfield, whereas no alteration occurred in ischemia-tolerant CA3/DG subfields, nor in postconditioning-treated brains. Interestingly, oligomerization of VDAC1 has been implicated in Alzheimer’s disease^20,22^. Overexpression of VDAC1 has also been found in Parkinson’s disease^21,34,35^, indicating that different pathogenesis involves in neurodegenerative diseases and stroke. Thus it is easy to understand that miR-7 is detrimental for stroke but actually beneficial for Parkinson’s disease treatment^35^. Likewise, either an increase or decrease in VDAC1 levels would have deleterious consequences, which contributes to different pathological processes.

The mechanism by which mitochondrial dysfunctions cause a decline of VDACs resulting in neuronal degeneration after ischemic stroke remains to be determined. Intracellular calcium overload contributes to postischemic neuronal death^36^. As known, VDACs are the only channels that transport Ca^2+^ across the OMM from the cytoplasm or the ER^37^. Our data suggest that VDAC1 is critical for the buffering of excessive intracellular Ca^2+^. It has been reported that cells with low levels of VDAC1 exhibit deficits in ATP production^38^. Our findings suggest that stabilized expression of VDAC1 is essential for ATP generation in the rat brain. Therefore, a postischemic decline of VDAC1 may suppress ATP-dependent Ca^2+^ transporters because of metabolism impairment rather than mitochondrial Ca^2+^ buffering capacity, which eventually results in an intracellular Ca^2+^ overload. Although the three VDAC isoforms in mammalian mitochondria share high sequence homology, these proteins have been found to exhibit different characteristics in their OMM localization^12^. The functional differences among the three VDAC isoforms remain to be determined.

Our results showed that brain ischemia and reperfusion caused the downregulation VDAC1 and VDAC3, whereas VDAC2 was least affected after global ischemia. Thus, there appear to be different regulatory mechanisms for the expression of the VDAC isoforms. MiR-7, which is highly expressed in hippocampal CA1 neurons^39^, has been found to be associated with VDAC1^35^. Here, we showed that miR-7 targeted both VDAC1 and VDAC3. An increase in miR-7 preceded the reduction of VDAC mRNAs and proteins after brain ischemia and the level of miR-7 was negatively correlated with the expression of VDAC1 and VDAC3, indicating that posttranscriptional regulation and translational repression by miR-7 are responsible for the postischemic decrease of VDAC1 and VDAC3. The regulatory mechanisms associated with abnormal expressions of miR-7 after I/R need to be addressed in future studies. Additional miRNAs that specifically target VDACs have been reported, including miR-29^40^ and miR-320a^41^. It was shown that glucose deprivation reduces miR-29 levels in cultured hippocampal astrocytes^42^. Anti-miR-320a leads to a reduction in the infarct volume after focal ischemia^43^. However, it remains undefined whether miR-29/miR-320a-VDAC1 axis is associated with ischemic brain damage.

Thus far, a desirable biomarker for the diagnosis of AIS is unavailable except for clinical and imaging tests. Our data showed that circulating miR-7 levels might provide a candidate for the early diagnosis and prognostic assessment of AIS. Higher rise of circulating miR-7 levels from patients than that from rats is likely age-related, indicating that circulating miR-7 levels could be a more sensitive biomarker for aged stroke patients, although further clinical studies are needed to validate its specificity and sensitivity. Importantly, we demonstrated a therapeutic effect of targeting miR-7 even after brain ischemia. Because reperfusion-induced delayed brain damage can occur after a stroke, it is important to develop therapeutically effective interventions for use after ischemic insults. Of note, the protective role of miR-7 inhibition probably contributes to the observed multiple targeting effects of this miRNA. Previously, it has been shown that miR-7 directly targets PARP^44^, Cox2^45^, α-synuclein^46^, Bax^47^, and the NLRP3 inflammasome^48^. However, the expression of these substrates seems unrelated to postischemic miR-7 levels, because postischemic increases of these molecules have been previously reported^49–53^. It would be interesting to investigate where miR-7 originates in the brain, and why miR-7 targets only a few specific substrates after brain ischemia.

Taken together, our findings provide insight into the molecular mechanism of ischemic postconditioning, which confers mitochondrial neuroprotection through stabilizing VDAC expression and maintaining bioenergy and calcium homeostasis. The data show that miR-7 is a promising target for the treatment of ischemic brain diseases, providing a novel and feasible therapeutic strategy for ischemic stroke.

## Materials and methods

### Experimental animals

All experimental procedures were approved by local Institutional Animal Care and Use Committee (Approval ID: SYXK (SU) 2016-0028). Male Sprague-Dawley (SD) rats (weighing 220–300 g) were kept on a normal light cycle at room temperature with free access to water and food. All animals were randomly allocated into different treat groups and results were assessed under blind conditions.

### Transient global ischemia models

Global ischemia was induced by four-vessel occlusion method as previously described^54^. Briefly, vertebral arteries of rats were electro-cauterized under anaesthetizing with 5% isoflurane for induction and 1.5%–2% isoflurane for maintenance followed by 24 h recovery and fast. The bilateral common carotid arteries were occluded with aneurysm clips for 15 min. The sham group was undergone the same procedures without carotid arteries occlusion. To conduct ischemic postconditioning, both carotid arteries were reclosed for another single 3 min after 10 min of reperfusion^8^. Rectal temperature was maintained at 37 ± 0.5 °C during and after the ischemic insult.

### Focal ischemia models

Focal cerebral ischemia was produced by intraluminal occlusion of the left middle cerebral artery for 90 min using a suture method in SD rats under anesthesia as mentioned above. After 24 h of ischemia and reperfusion, rat brains were harvested and frozen for 20 min at −20 °C. The rat brains were cut into coronal sections (2 mm) and then stained with 2, 3, 5-tri-phenyltetrazolium chloride (TTC; 2% in 0.1 M phosphate buffer. Cinontech Co. Ltd, Beijing, China) for 30 min at 37 °C followed by fixing with 4% paraformaldehyde. ImageJ software was used to estimate the infarct volume as previously described^55^. Neurological deficit scores were used to score the neurological motor function^56^ as follows: 0, no neurological deficit; 1, failure to extend the contralateral forepaw fully; 2, circling to the contralateral; 3, failing to the contralateral; and 4, no spontaneous movement. Animals with scores 1–3 were included in the experiments.

### Stereotaxic injection

The coordinates used for rat hippocampal CA1 subfield injections were anteroposterior, 3.6 mm, lateral, 2.0 mm, and depth, 4.0 mm from bregma. Coordinates used for cerebral lateral ventricle injections were anteroposterior, 0.8 mm, lateral, 1.5 mm, and depth, 3.5 mm from bregma. For VDAC1 knockdown, si-VDAC1 (45 pmol; GenePharma, Shanghai, China) was injected into rat right hippocampal CA1 regions 50 min after global ischemia. For VDAC2 or VDAC3 knockdown, a pool of 3 different siRNA duplexes of VDAC2 or VDAC3 was used. Polyethylenimine (PEI) (25 kDa branched, Sigma-Aldrich, St. Louis, MO, USA) was diluted to 0.1 mM (PH 7.0) using 5% glucose as described previously^57^. The mixture of equivalent volume PEI and siRNA pool (75 pmol, GenePharma) were equilibrated for 15 min and then injected into hippocampal CA1 region 50 min after global ischemia. For miR-7 inhibition in vivo, Anti-7 or negative control (NC) (0.1 nmol for global ischemia; 1 nmol for focal ischemia; GenePharma) was delivered into rat right cerebral lateral ventricles 50 min after global ischemia or 40 min after MCAO onset, respectively.

si-VDAC1: 5′ GAGGGAGCAUAUCAACCUGTT 3′;

si-VDAC2: 5′ CCGGUAUGCCAACGGCCAATT 3′; 5′ GCAAUUGAAGACCAGAUUUTT 3′; 5′ CCAUGGGUCAGCCGUCUUUTT 3′;

si-VDAC3: 5′ GCUGCCAAGGAUGUCUUUATT 3′; 5′ GCAACCUAGAGACCAAAUATT 3′; 5′ CCAUCUACCAGAGAGUUAATT 3′;

si-NC: 5′ UUCUCCGAACGUGUCACGUTT 3′.

Anti-7: 5′ ACAACAAAAUCACUAGUCUUCCA 3′;

NC: 5′ UUGUACUACACAAAAGUACUG 3′.

### Brain tissue sampling and immunoblots

For samples from global ischemia, the hippocampal CA1 or CA3/DG subfield and for samples from focal ischemia, regions predesignated as “core” and “penumbra”^58^ were harvested and rapidly frozen in liquid nitrogen. Each brain tissue sample was homogenized in ice-cold homogenization buffer containing protease inhibitors^54^. HT22 cell lines were lysed with homogenization buffer containing protease inhibitors. Tissue homogenates or cell lysates were centrifuged at 800 × *g* for 15 min at 4 °C and the supernatants were collected. Lowry method was used for protein quantitation. Proteins solubilized in 4 × Laemmli sample buffer were to SDS-PAGE and then transferred onto nitrocellulose membranes. After blocking with 3% bovine serum albumin for 3 h at room temperature, the membranes were probed with various primary antibodies overnight at 4 °C. Anti-VDAC1 (1:1000; cat. #MABN504; Millipore, Billerica, MA, USA); anti-VDAC2 (1:2000; cat. #PA1-958; Thermo Fisher Scientific, Scotts Valley, CA, USA); anti-VDAC3 (1:1000; cat. #AV35180, Sigma-Aldrich); anti-COX4 (1:3000; cat. #4844; Cell Signaling Technology, Danvers, MA, USA); anti-mitofusin 1 (1:2000; cat. #ab57502, Abcam, Inc, Cambridge, UK) and anti-Actin (1:5000; cat. #4970; Cell Signaling Technology). After washing with Tris-buffered saline with 0.1% Tween-20, the membranes were incubated with corresponding secondary antibodies conjugated with horseradish peroxides for 1 h. Detection was conducted by the ECL kit (Millipore) according to the manufacturer’s instructions. Proteins bands were scanned and analyzed with Quantity One 1-D Analysis Software (Bio-Rad, Hercules, CA, USA).

### qReal-time PCR for mRNA and miRNA detection

The hippocampal CA1 or CA3/DG subfield from global ischemia, and penumbra region from focal ischemia were harvested and rapidly frozen in liquid nitrogen. Total RNA from brain and blood samples was extracted with Trizol® reagent (Invitrogen Life Science, Carlsbad, CA, USA). The concentration and integrity of the RNA were determined using NanoDrop^™^ 2000 (Thermo Fisher Scientific). RNA was reverse transcribed utilizing the PrimeScript^™^ RT reagent kit (DRR037; TaKaRa Bio, Beijing, China) according to the manufacturer’s instructions. Quantitative PCR was carried out using SYBR Premix Ex Taq II (DRR820; TaKaRa) and the StepOne™ Plus real-time PCR system (Applied Biosystems, Foster City, CA, USA). Primers for VDAC1 were 5′ GACAACACCCTGGGCACTG 3′ (forward) and 5′ CACAGCCCAGGTTGATATG 3′ (reverse); Primers for VDAC2: 5′ GAATGTTGTGTACCGGTATGC 3′ (forward) and 5′ CCAGTGTCTGTATTAGATGAG 3′ (reverse); Primers for VDAC3: 5′ GTAACTACGGGCTCATCTTCAC 3′ (forward) and 5′ CGTCAGTTTCAACCCTTCAGCC 3′ (reverse); Primers for COX4: 5′ TCGCTGAGATGAACAAGG 3′ (forward) and 5′ ATGGAAGCCGATGAAGAAC 3′ (reverse); Primers for Actin: 5′ CCCATCTATGAGGGTTACGC 3′ (forward) and 5′ TTTAATGTCACGCACGATTTC 3′ (reverse).

For assessment of VDAC and COX4 mRNA expression, Actin mRNA was used as an internal reference. For evaluation of miRNA expression, U6 served as an internal control. Primers for miR-7 were 5′ GTCGTATCCAGTGCAGGGTCCGAGGTATTCGCACTGGATACGACACAACAA 3′ (RT primer); 5′ CTGGAGTGGAAGACTAGTGATT 3′ (forward) and 5′ GTGC AGGG TCCG AGGT 3′ (reverse); Primers for U6: 5′ AAAATATGGAACGCTTCACGAATTTG 3′ (RT primer); 5′ CTCGCTTCGGCAGCACATATACT 3′ (forward) and 5′ ACGCTTCACGAATTTGCGTGTC 3′ (reverse). The 2^-ΔΔCT^ method was used to calculate the fold changes.

### Quantification of mtDNA copy number

Total DNA was isolated from rat hippocampal CA1 subfield after reperfusion according to the manufacturer’s instructions (TIANamp Genomic DNA Kit, TIANGEN, Beijing, China). mtDNA was amplified using primers specific for the mitochondrial cytochrome c oxidase subunit 2 (*Cox2*) gene and normalized to genomic DNA by amplification of the *Rps18* nuclear gene. Primers for *Cox2* were 5′ ATAACCGAGTCGTTCTGCCAAT 3′ (forward) and 5′ TTTCAGAGCATTGGCCATAGAA 3′ (reverse); for *Rps18*: 5′ TGTGTTAGGGGACTGGTGGACA 3′ (forward) and 5′ CATCACCCACTTACCCCCAAAA 3′ (reverse).

### Histological assessment of hippocampal damage

Histological assessment of hippocampal damage was performed in a blinded manner as described previously^8^. Rats were perfusion-fixed with 0.9% saline and 4% paraformaldehyde under anesthesia after global ischemia followed by 5 d of reperfusion. Brains were removed and further fixed with the same fixation solution at 4 °C overnight. Postfixed brains were embedded with paraffin and then coronal sections (6 μm thick) were prepared using a microtome (Leica, Wetzlar, Germany). The paraffin-embedded brain sections were deparaffinized with xylene and rehydrated in a graded concentration of ethanol, followed by washing with distilled water. The sections were stained with 0.1% Cresyl Violet (Sigma-Aldrich) to assess the neuronal survival in the hippocampus. The numbers of surviving hippocampal CA1 neurons per 1 mm length were counted as the neuronal density.

### HPLC analysis for ATP detection

The hippocampal CA1 samples were immediately homogenized with 0.6 M perchloric acid. The homogenates were centrifuged at 5000 × *g* for 10 min at 4 °C and neutralized with 1 M KOH and then centrifuged at 5000 × *g* for 10 min at 4 °C. Filtered supernatants (0.22 μm filter, Millipore) were stored at −80 °C until processing. A 20 μl supernatant was injected to determine ATP levels by HPLC (Hypersil ODS2 column: 4.6 mm × 150 mm, 5 μm; Elite Analytical Instruments Co. Ltd, Dalian, China) using 254 nm wavelength; Mobile phase was 84 mM phosphate buffer (61 mM NaH_2_PO_4_ and 23 mM Na_2_HPO_4_ buffer solution, PH 6.5) and methanol (99.9: 0.1) and flow rate was 1.0 ml/min. Column temperature: 30 °C. ATP levels were determined by ATP standard curve (ATP standard, Roche, Mannheim, Germany) and presented as fold changes relative to the sham group.

### ROS assay

The levels of ROS were determined by dichlorofluorescein diacetate (DCFH-DA) as described previously^59^. The hippocampal CA1 region was homogenized using 0.1 M PBS (1: 20) followed by centrifugation at 1000 × *g* 10 min at 4 °C. The level of ROS was measured with ROS assay kit (Jiancheng Bio-engineering Institute, Nanjing, China). Briefly, 190 μl supernatant mixed with 10 μl DCFH-DA or 10 μl PBS were incubated at 37 °C for 30 min in 96-well plates protected from light. Detection was conducted in GloMax Discover Multimode Microplate Reader (Promega, Sunnyvale, CA, USA) using 480 nm for excitation and 520 nm for emission. Lowry method was used for protein quantitation of samples. The ROS levels are presented as fluorescence intensity/mg protein.

### VDAC1 knockdown by a gene-editing system

HT22 mouse hippocampal cell lines were cultured in DMEM plus 10% fetal bovine serum in a humidified incubator with 5% CO_2_ and 95% air at 37 °C. Genomic knockdown of VDAC1 was carried out using CRISPR Vector pX330 plasmid (Plasmid # 423230; Addgene, Cambridge, MA, USA) encoding an optimized hSpCas9 gene and the vdac1-specific guide RNA. The specific guide RNA sequence was as follows: 5′ TCGGCGTATGTGGGAGGCACGG 3′. HT22 cells transfected with the targeting plasmid were screened to collect single cell colonies, and then identified by sequencing. Cell colonies with frame-shift mutations in one of the VDAC1 alleles were considered as knockdowns. Immunoblot analyses were performed to verify the knockdown events.

### Calcium imaging

Cytosolic calcium changes in response to ionomycin (Sigma-Aldrich) were monitored by GCaMP6f, a genetically encoded calcium indicator (A gift from Prof. Wei Xiong, School of Life Science, Tsinghua University). The pGP-CMV-GCaMP6f plasmid (0.5 μg/well) was transfected into HT22 cells in 24-well plates using Lipofectamine^®^ 2000 (Invitrogen Life Science) according to the manufacturer’s instructions. G418 (Gibco-BRL, Gaithersburg, MD, USA; 750 μM) was used for 72 h to screen the successfully transfected HT22 cells. HT22 cells were imaged after ionomycin stimulus (2 μM in Tyrode’s solution). Tyrode’s solution contains 130 mM NaCl, 4 mM KCl, 2 mM CaCl_2_, 1 mM MgCl_2_, 10 mM glucose, and 10 mM HEPES (pH 7.2 adjusted with NaOH) The green fluorescent signal was acquired by fluorescence microscopy (Olympus, Tokyo, Japan).

Intracellular calcium was also determined by a Ca^2+^-sensitive fluorescent dye, Fura-2/AM (Sigma-Aldrich), as previously described^54^. Briefly, VDAC1^+/+^ and VDAC1^+/−^ HT22 cell lines were washed with Tyrode’s solution twice and incubated with Fura-2/AM (2 μM in Tyrode’s solution) at 37 °C for 30 min protected from light followed by being washed twice again. Lambda DG-4 Ultra High Speed Wavelength Switcher was used to alternately excite 340 nm and 380 nm for the ratiometric measurements of Fura-2 fluorescence. High-speed EMCCD camera was used to collect emitted fluorescence at 510 nm, and MetaFluor software was used to record the ratio of fluorescence at 340 nm–380 nm. The fluorescent ratio was recorded every 10 seconds. Ionomycin (2 μM in Tyrode’s solution) or caffeine (20 mM in Tyrode’s solution) was added to induce calcium fluctuations.

Mitochondrial Ca^2+^ in permeabilized HT22 cell lines was determined using Rhod-2/AM (4 μM, Dojindo, Shanghai, China), as described previously^60^. The incubation and wash process were similar to cytosolic Ca^2+^ measurement using Tyrode’s solution. After loading with Rhod-2/AM, the permeabilization solution, 0.005% saponin (Sigma-Aldrich) in Ca^2+^-free internal solution (100 mM potassium acetate, 15 mM KCl, 0.35 mM EGTA, 0.75 mM MgCl_2_, 10 mM HEPES, pH adjusted to 7.2 with KOH) was used 1 min to remove cytosol-localized Rhod-2 followed by being washed twice with Ca^2+^-free internal solution. When starting image acquisition, Ca^2+^-replete internal solution (2 μM) was used. The concentration of Ca^2+^-replete internal solution was calculated by MaxChelator program (maxchelator.stanford.edu). The fluorescent signal was acquired by fluorescence microscopy (Olympus). The amplitude of change of mitochondrial Ca^2+^ was shown as the ratio of fluorescence value (*F*) after adding Ca^2+^ in region of interest to the fluorescence prior to Ca^2+^ addition (*F*_0_).

### Blood samples

The clinical experiment was approved by the Ethics Committee of Xuzhou Medical University. All written informed consents were acquired from patients or healthy control participants prior to inclusion in the study. Blood samples were obtained from initial episode patients within 24 h after acute ischemic onset from December 2016 to June 2017. Ages ranged from 50 to 75 years of age. Computerized tomography or magnetic resonance imaging was used to confirm the diagnoses. The National Institutes of Health Stroke Scale scores of all ischemic stroke patients ranged from 4 to 15. Exclusion was made according to the following criteria: secondary stroke or derived from other types such as subarachnoid hemorrhage, cerebrovascular malformation, and brain tumor; recurrent stroke; acute infectious diseases; kidney and liver system diseases; carcinoma; inflammation and autoimmune disease; and mental disorders such as schizophrenia or depression. Healthy control participants were recruited from routine health checkups with matched ages of stroke patients. All healthy controls met the exclusion criteria described above, and precluded stroke and any risk factors as previously reported^61^. Peripheral venous blood was collected with EDTA anticoagulant tubes and stored at −80 °C for further processing.

### Dual-luciferase reporter assay

The 3′-UTR regions (45 nucleotide sequence containing binding sites of miR-7, or the full length) of VDACs or mutated forms (Sangon Biotech, Shanghai, China) were inserted into the pmirGLO vector (Promega) with *Sac* I and *Xba* I at the 3′ end of the luciferase gene to construct the reporter plasmids pmirGLO-VDACs-WT or pmirGLO-VDACs-MUT. HEK293 cells or HT22 cells were seeded in 24-well plates and transfected with 50 nM of miR-7 mimic (5′ UGGAAGACUAGUGAUUUUGUUGU 3′) or miR-NC (5′ UUCUCCGAACGUGUCACGUTT 3′) together with 125 ng luciferase reporter plasmid. PEI (Sigma-Aldrich) was used for transfection and cells were harvested at 24 h after transfection. Luciferase activity was measured with Dual-Luciferase Reporter Assay kit (Promega) according to the manufacturer’s instructions.

### Statistics

Unless otherwise indicated, the results are shown as mean ± SD. Data were obtained from at least three independent assessments for each experiment. Statistical analysis was conducted with SPSS software (SPSS, Chicago, IL, USA) using two-tailed Student’s *t*-test or unpaired *t*-test with Welch’s correction between two groups. For comparison among groups, one-way ANOVA followed by Dunnett or Dunnett’s T3 post-hoc tests was used. For assessing fluctuations of [Ca^2+^] after ionomycin or caffeine treatment or mitochondrial calcium, results are shown as mean ± SEM and Two-way ANOVA was used. Significant differences were values of *P* *<* 0.05. All analytics were used after checking for normality and homogeneity of variance.

## Electronic supplementary material

Figure S1

Figure S2

Figure S3

Figure S4

Supplementary Information of ischemic postconditioning confers cerebroprotection by stabilizing VDACs after brain ischemia
