# Supplementary figures and images for: Ischemic postconditioning confers cerebroprotection by stabilizing VDACs after brain ischemia
Source: Cell Death Dis. 2018 Oct 10;9(10):1033. doi: 10.1038/s41419-018-1089-5 (PMC6180002; doi:10.1038/s41419-018-1089-5)

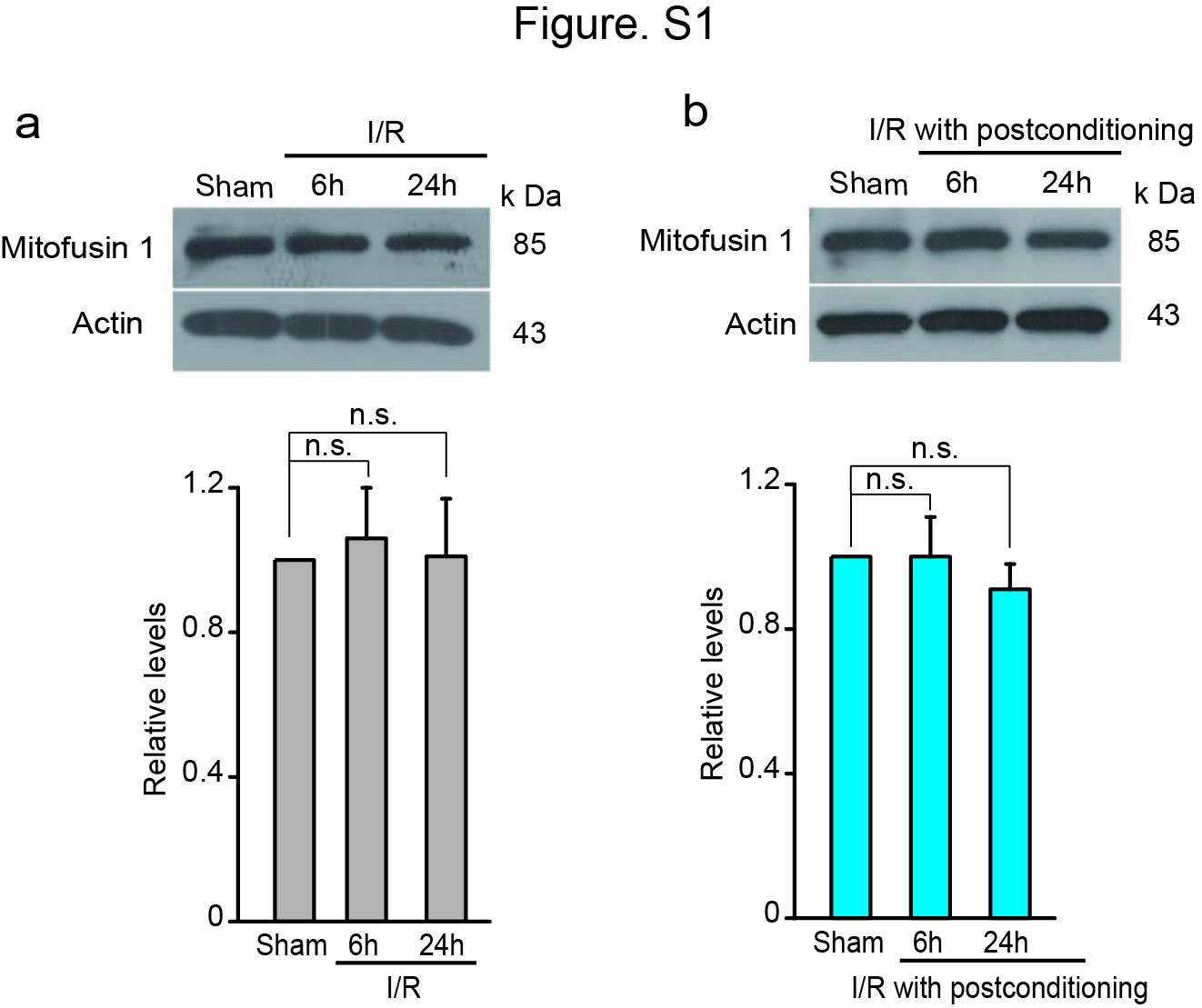

Supplement: Supplementary file 1 — Figure S1 [file 41419_2018_1089_MOESM1_ESM.jpg]

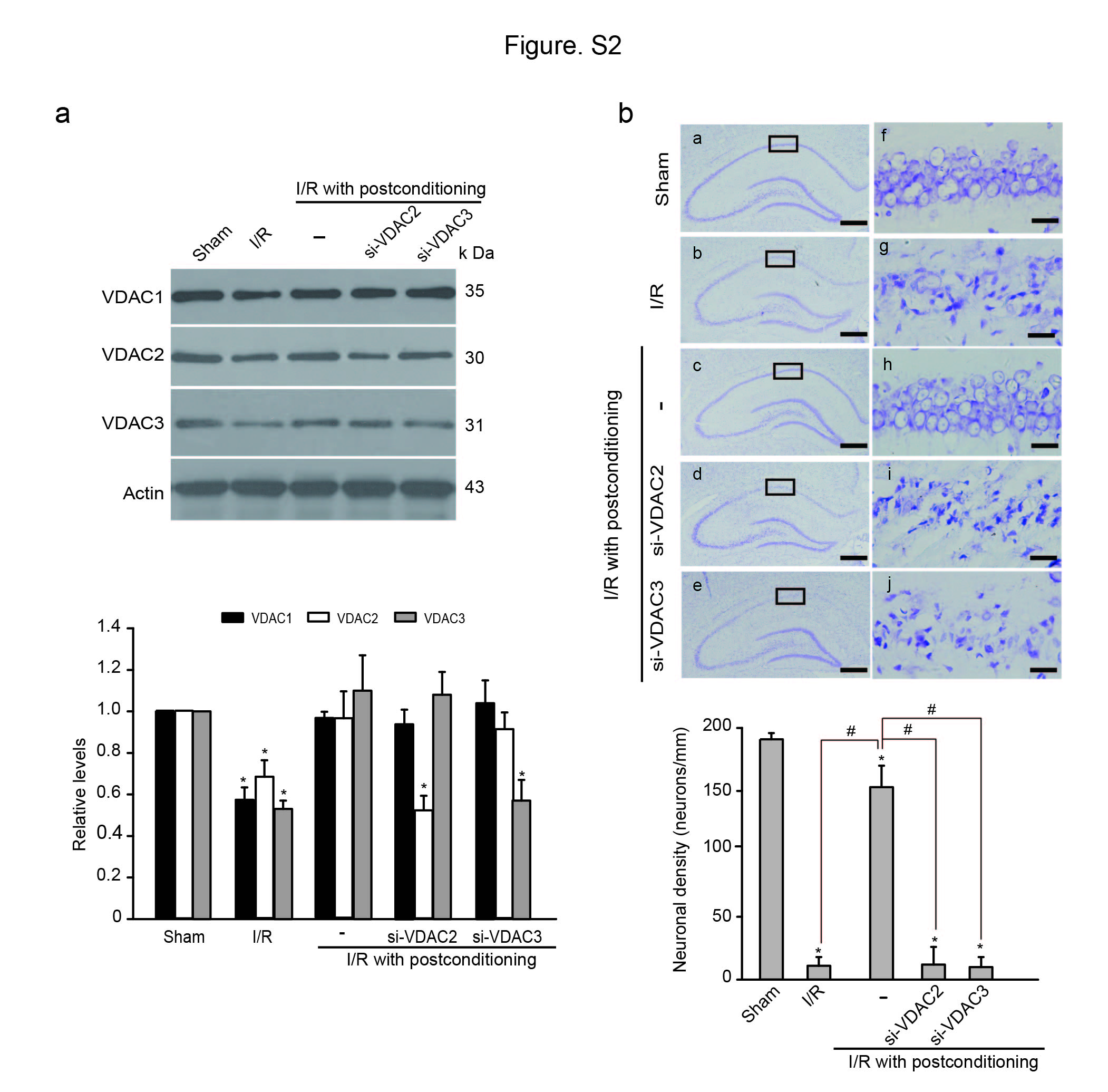

Supplement: Supplementary file 2 — Figure S2 [file 41419_2018_1089_MOESM2_ESM.jpg]

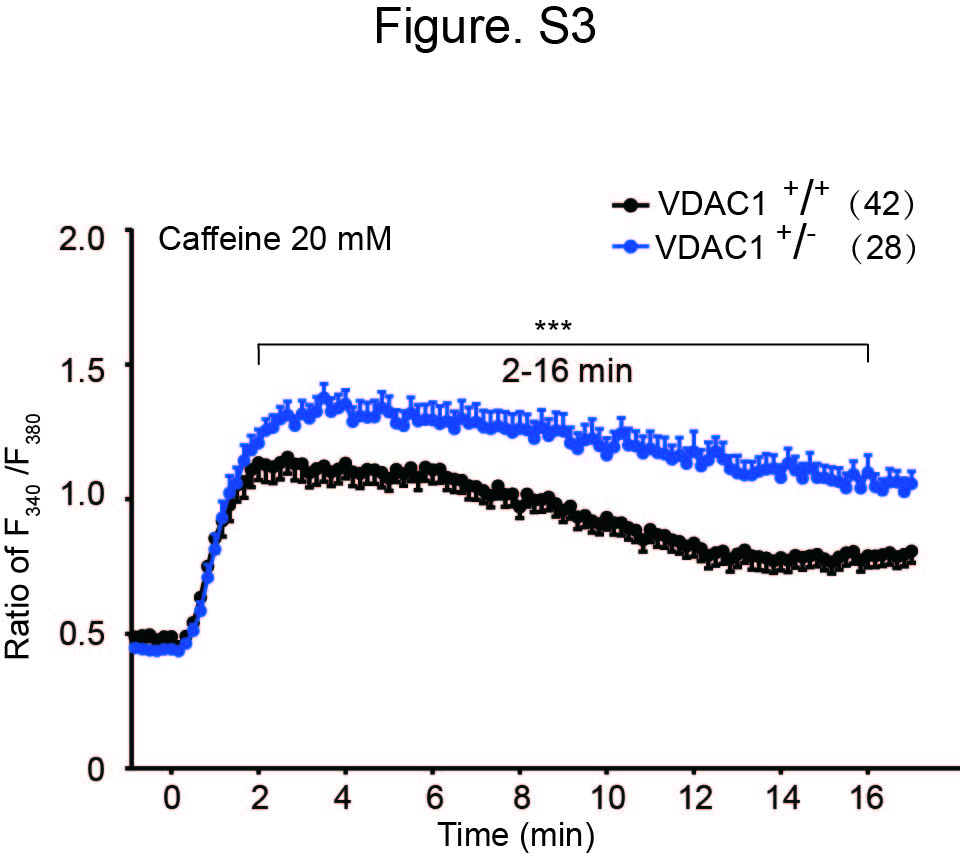

Supplement: Supplementary file 3 — Figure S3 [file 41419_2018_1089_MOESM3_ESM.jpg]

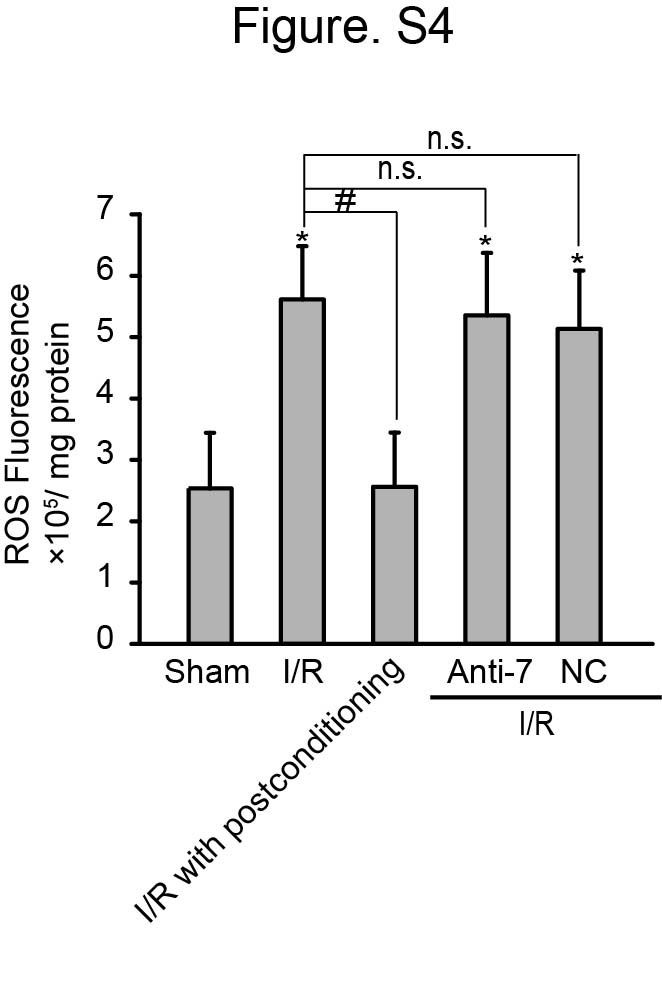

Supplement: Supplementary file 4 — Figure S4 [file 41419_2018_1089_MOESM4_ESM.jpg]
