# Supplementary material for: Ischemic postconditioning confers cerebroprotection by stabilizing VDACs after brain ischemia
Source: Cell Death Dis. 2018 Oct 10;9(10):1033. doi: 10.1038/s41419-018-1089-5 (PMC6180002; doi:10.1038/s41419-018-1089-5)
Supplement: Supplementary file 5 — Supplementary Information of ischemic postconditioning confers cerebroprotection by stabilizing VDACs after brain ischemia [file 41419_2018_1089_MOESM5_ESM.docx]

**Ischemic postconditioning confers cerebroprotection by stabilizing VDACs after brain ischemia**

**Supplementary Information**

**Fig. S1 The expression of mitofusin 1 has no alteration in rat hippocampal CA1 subfields after brain ischemia.** Immunoblots and quantification of mitofusin 1 after ischemia and reperfusion (I/R) without (a) or with ischemic postconditioning (b) in the CA1 subfield (*n* = 3 rats per group); Relative levels of mitofusin 1 were normalized to respective sham groups. Actin was used as a loading control. Data are shown as the mean ± SD of three independent experiments. n.s., not significant; One-way ANOVA.

**Fig. S2 VDAC2 or VDAC3 knockdown abolishes the neuroprotection of ischemic postconditioning in rat hippocampal CA1 subfields.** **a** Immunoblots and quantification of VDACs in the hippocampal CA1 region 24 h after ischemia and reperfusion (I/R) in rats treated with si-VDAC2 or si-VDAC3 after postconditioning (*n* = 3 rats per group); Relative levels were normalized to respective sham groups. Actin was used as a loading control. Data represent the mean ± SD of three independent experiments; ^＊^*P* < 0.05 versus the sham group; One-way ANOVA. **b** Upper, Nissl stained hippocampal sections 5 d after I/R without or with postconditioning in rats treated with si-VDAC2 or si-VDAC3. Scale bar, 500 μm from a to e. Scale bar, 20 μm from f to j. Lower, quantification of the surviving neurons in the hippocampal CA1 subfield for each group (*n* = 5 rats per group). Data are the mean ± SD; ^＊^*P* < 0.05 versus the sham group; ^#^*P* < 0.05 versus the I/R with postconditioning group; One-way ANOVA.

**Fig. S3 Intracellular calcium buffering capacity is damaged in VDAC1^+/-^ HT22 cells.** [Ca^2+^]_i_ fluctuations induced by caffeine (20 mM) treatment in VDAC1^+/+^ or VDAC1^+/-^ HT22 cells were detected by Fura-2/AM. Data represent the mean ± SEM (*n* = 42 for VDAC1^+/+^ HT22 cells; *n =* 28 for VDAC1^+/-^ HT22 cells). ^＊＊＊^*P* < 0.01 (2-16 min after caffeine treatment); Two-way ANOVA.

**Fig. S4 Ischemic postconditioning reduces the levels of ROS**. Quantification of ROS in the hippocampal CA1 region 24 h after ischemia and reperfusion (I/R) without or with postconditioninging, Anti-7, or negative control (NC) treatment. Data are shown as the mean ± SD (*n* = 5 rats per group). ^＊^*P* < 0.05 versus the sham group; ^#^*P* < 0.05 versus the I/R; n.s., not significant; One-way ANOVA.
